# Supplementary material for: Complexome profiling of the Chlamydomonas psb28 mutant reveals TEF5 as an early PSII assembly factor
Source: Plant Cell. 2025 Mar 17;37(6):koaf055. doi: 10.1093/plcell/koaf055 (PMC12164590; doi:10.1093/plcell/koaf055)
Supplement: koaf055_Supplementary_Data [file koaf055_supplementary_data.zip › psb28_interactive.zip!/plots/intensity/qualityPlots/peptides/MDN4_Cre12.g483950.t1.2.html]

### Cre12.g483950.t1.2

Trivial name: MDN4  
SeqCoverage [median]: 59.20  
Mass: 35.14 kDa  
  
 

| Sequence | MissedCleavage | Mass [Da] | wtIntensity | muIntensity | Score |
| --- | --- | --- | --- | --- | --- |
| AGLKAMMPELLASIEK | 1 | 1700.92 | 4279.61 | 19653.21 | 108.34 |
| AKTFYAEK | 1 | 956.50 | 1228.81 | 0.00 | 83.21 |
| AMMPELLASIEK | 0 | 1331.68 | 91798.36 | 170253.96 | 166.66 |
| ATMSAEVLDALTK | 0 | 1348.69 | 998.52 | 0.00 | 101.48 |
| ATMSAEVLDALTKR | 1 | 1504.79 | 119300.07 | 217860.77 | 201.08 |
| DDLFKINAGIVR | 1 | 1359.75 | 2389.44 | 3305.48 | 103.91 |
| GFDKDGLAEALR | 1 | 1290.66 | 5273.93 | 9745.56 | 91.94 |
| IHDLGPLSDYEK | 0 | 1385.68 | 378.30 | 2930.36 | 49.45 |
| INAGIVR | 0 | 741.45 | 45590.53 | 85948.54 | 152.26 |
| KMGVYDK | 1 | 839.42 | 774.48 | 0.00 | 93.56 |
| KPGMTRDDLFK | 2 | 1306.67 | 3733.86 | 4079.34 | 54.26 |
| KVAVLGAAGGIGQPLSMLMK | 1 | 1940.10 | 20555.06 | 26056.61 | 102.69 |
| KVMGVTTLDVVR | 1 | 1316.75 | 36397.59 | 49525.38 | 136.38 |
| LSTEGVDK | 0 | 847.43 | 2286.32 | 9178.64 | 91.06 |
| LSTEGVDKIHDLGPLSDYEK | 1 | 2215.10 | 33482.96 | 59221.37 | 132.01 |
| MGVYDKR | 1 | 867.43 | 1395.76 | 0.00 | 82.31 |
| MNSQVSSLSLYDIAGTPGVAADVSHINTK | 0 | 2974.47 | 13526.84 | 20504.34 | 121.75 |
| NGLDVASVDVPVVGGHAGVTILPLFSQATPK | 0 | 3057.65 | 1744.34 | 0.00 | 49.61 |
| RTQDGGTEVVQAK | 1 | 1387.71 | 26930.46 | 16989.51 | 131.79 |
| TFYAEK | 0 | 757.36 | 11785.08 | 20117.86 | 144.90 |
| TQDGGTEVVQAK | 0 | 1231.60 | 84201.84 | 89652.37 | 248.05 |
| VAVLGAAGGIGQPLSMLMK | 0 | 1812.00 | 13892.79 | 36547.42 | 177.42 |
| VKLSTEGVDK | 1 | 1074.59 | 1279.74 | 2630.07 | 79.20 |
| VKLSTEGVDKIHDLGPLSDYEK | 2 | 2442.26 | 4965.65 | 12956.25 | 70.31 |
| VMGVTTLDVVR | 0 | 1188.65 | 61369.52 | 122445.07 | 205.83 |
|  |  |  | 589559.9 | 979602.1 |  |
